# Emergent effects of global change on consumption depend on consumers and their resources in marine systems

**DOI:** 10.1073/pnas.2108878119

**Published:** 2022-04-21

**Authors:** Tye L. Kindinger, Jason A. Toy, Kristy J. Kroeker

**Affiliations:** ^a^Department of Ecology and Evolutionary Biology, University of California, Santa Cruz, CA 95060

**Keywords:** trophic interactions, multiple stressors, warming, ocean acidification, meta-analysis

## Abstract

A better understanding of how environmental change will affect species interactions would significantly aid efforts to scale up predictions of near-future responses to global change from individuals to ecosystems. To address this need, we used meta-analysis to quantify the individual and combined effects of ocean acidification (OA) and warming on consumption rates of predators and herbivores in marine ecosystems. Although the primary studies demonstrated that these environmental variables can have direct effects on consumers, our analyses highlight high variability in consumption rates in response to OA and warming. This variability likely reflects differences in local adaptation among species, as well as important methodological differences. For example, our results suggest that exposure of consumers to OA reduces consumption rates on average, yet consumption rates actually increase when both consumers and their resource(s) are concurrently exposed to the same conditions. We hypothesize that this disparity is due to increased vulnerability of prey or resource(s) in conditions of OA that offset declines in consumption. This hypothesis is supported by an analysis demonstrating clear declines in prey survival in studies that exposed only prey to future OA conditions. Our results illustrate how simultaneous OA and warming produce complex outcomes when species interact. Researchers should further explore other potential sources of variation in response, as well as the prey-driven component of any changes in consumption and the potential for interactive effects of OA and warming.

## Significance

Understanding the effects of global change on species interactions is important for predicting emergent ecosystem changes. Although environmental change can have direct effects on consumers, it is unclear if consumption will change in any generalizable way when both the consumer and resource(s) are exposed to future conditions. Using meta-analysis, we show high variability in consumption rates in response to ocean acidification and warming, indicating conclusions that suggest consumption will generally increase or decrease in a future ocean are premature. We also demonstrate how the interpretation is dependent on whether only the consumer or both the consumer and its resource(s) are exposed to future conditions. Based on these findings, we provide a road map for future research in this area.

Numerous studies have demonstrated direct effects of ocean acidification (OA) and warming on organismal physiology and performance (1, 2), yet forecasting the emergent ecological effects of environmental change on communities remains a challenge due to the complexity of interactions between species (3–5) and stressors (1, 2, 6–8) in functioning ecosystems. Species interactions have the potential to drive shifts in communities (5, 9–11) or buffer them (5, 12, 13) from environmental change. For example, environmentally mediated increases in growth of some algal species can lead to ecosystem shifts if they outcompete or overgrow other species (14). However, environmentally mediated increases in consumption rates of key herbivores have the potential to limit this overgrowth of algae and the associated community shift (13). Indeed, authors of several of the studies that have revealed potential emergent effects of OA (14–16) or warming (17, 18) on entire marine communities attributed the observed responses at least in part to changes in species interactions. In addition, pronounced shifts in species assemblages and ecosystems during natural, large-scale warming events are often linked to modified species interactions (19, 20). Establishing general patterns of environmental control on species interactions has thus been proposed as a promising avenue for scaling up the effects of environmental change from individuals to ecosystems (9, 21–24).

Environmentally mediated changes in trophic interactions may be especially important in determining the effects of global change on ecosystems, due to their potential to have cascading effects on community structure (25, 26). OA (27, 28) and warming (29–31) are generally predicted to alter consumers’ energetic demands, although the effects on consumption will depend on the shape of the performance curves, how close current environmental temperatures are to their performance optima, the magnitude of the environmental change, and their energy allocation strategies (25). The effects of OA on consumption are more likely to vary among taxa with different traits than the effects of warming, which universally affects metabolism. For example, the effects of OA can vary with the degree of calcification or the level of mobility of a given species (1, 2). However, the ability of consumers in nature to sufficiently compensate for changes in energetic demands associated with either OA or warming through altered consumption will also depend on their prey or resources (32–34). For example, an increase in a consumer’s energetic demand could be met through increased ingestion of a given resource. Shifts in the escape response, production of physical or chemical deterrents, or the size or biomass of the resource species driven by environmental change, however, can mediate the outcome (4). Thus, deciphering the environmental controls on trophic interactions requires analysis of both the consumer- and resource-driven components of predation and herbivory in future conditions.

In most studies that assess the effects of OA, warming, or both on the consumption rates of marine species, researchers use controlled laboratory experiments that are amenable to meta-analysis. In these experiments, a consumer or resource is most often exposed to current and future environmental conditions for a period of days to months. Thus, the effects measured are primarily plastic and represent an organism’s ability to acclimate physiologically or behaviorally. Often, the consumer is held in treatment conditions and given prey or a resource that has not been acclimated to the experimental conditions (defined here as consumer-only experiments). In contrast, more complex studies (e.g., multispecies mesocosms or studies focused on species interactions or community-level responses) tend to include both the consumer and its resource(s) in the experimental conditions. While the responses in these experiments still represent the physiological or behavioral acclimation of the species involved, these multispecies experiments capture potential emergent effects of environmental change on species interactions that result from the direct effects on both the consumer and the resource. Prior meta-analyses have shown that there can be important variation in the temperature sensitivity of different ecological rates, such as attack rates and escape rates that can influence the emergent effects based on variation in sensitivity among trophic roles (30). Similarly, OA has been shown to affect both predator and prey detection and behavior (35–37), as well as algal traits that could affect herbivory, such as nutritional status or chemical deterrents (37). Most rare are those studies that expose only prey or resources to experimental conditions and then test their vulnerability to predation using a predator or herbivore that is not acclimated to the experimental conditions being tested. In contrast to the consumer-only experiments, these “resource-only” experiments capture how environmental change may affect the vulnerability or palatability of species that serve as resources.

Experiments also vary in the complexity of environmental manipulation. Although OA and warming are happening in concert in nature, many early studies focused on the biological effects of OA or warming in isolation. As global change biology has progressed, the focus has shifted toward multifactor studies that incorporate both OA and warming in combination (e.g., factorial experiments). These studies are critically important for forecasting emergent effects, as the combined effects of OA and warming may not be additive (1, 2). This may be especially important if OA and warming have different modes of action on marine organisms (38). Synergisms, in which the effects of OA and warming exacerbate one another, have gained the most attention because of their potential to cause dramatic ecological shifts. However, even with different modes of action, one environmental-change factor may primarily drive an organism’s overall response, leading to unexpected outcomes.

We conducted a systematic review (*SI Appendix*, Fig. S1 and Dataset S1) to assemble a database of published studies (Dataset S2) and conduct a meta-analysis quantifying the individual and combined effects of OA and warming on consumption rates, testing for variation between predator–prey and herbivore–resource interactions. We also tested for variance in the individual and combined effects of each environmental variable on different trophic roles (i.e., studies that exposed only consumers or only resources to treatment conditions prior to measuring consumption) to provide insight on their relative importance in the overall response of predation and herbivory rates in future conditions. We then tested whether taxonomic groups or life stages of interacting organisms explain any remaining variance among underlying studies to aid interpretation and identify gaps in our knowledge that need to be addressed to move the field forward. We also quantified the effects of OA and warming on prey survival in resource-only experiments, as well as the effects on consumer preference of prey or resources raised in ambient or future conditions. Finally, to quantitatively address the potential for nonadditive effects of the combined exposure to OA and warming, we calculated the individual and interactive effects of OA and warming on consumption rates for the subset of studies that factorially manipulated both variables.

## Results and Discussion

**Fig. 1.**
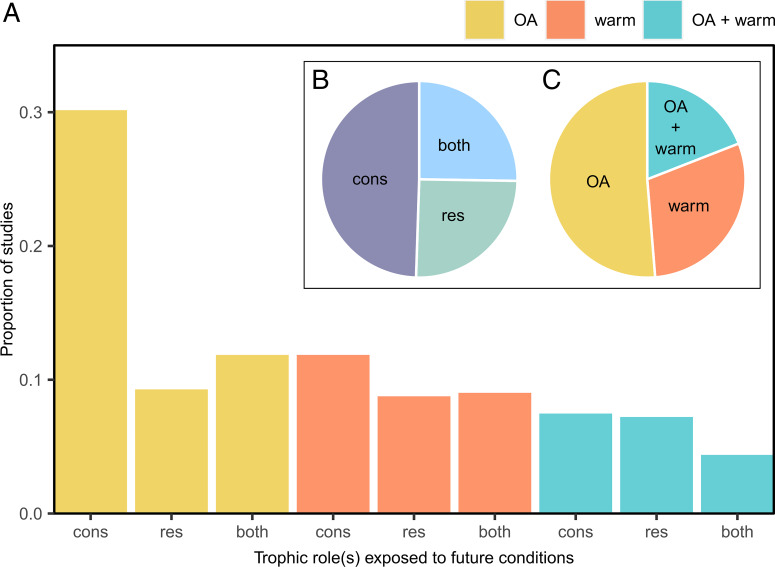
Studies of the individual and combined effects of OA and warming (warm) on consumption included in the analyses, as identified by the systematic review. (*A*) The proportion of studies that exposed only consumers (cons), only resources (res), or both consumers and their resources (both) to experimental conditions for each environmental-change scenario considered. (*B* and *C*) Insets highlight the total proportion of studies focused on different trophic roles (*B*) and environmental-change drivers (*C*).

Using a systematic review (*SI Appendix*, Fig. S1), we synthesized the results of 434 studies from 133 published articles on the effects of OA, warming, or OA and warming combined on consumption rates of marine species. Half (51.3%) of these studies examined the effects of OA in isolation, 29.6% of studies focused on the isolated effects of warming, and 19.1% of studies tested the combined effects of OA and warming (Fig. 1). Approximately half of all of these studies were consumer-only studies (i.e., only the consumer was treated), with resource-only and consumer + resource studies being similarly represented (Fig. 1). The category with the fewest studies was the consumer + resource studies in combination with OA and warming conditions, which are the most realistic conditions for predicting emergent effects of future environmental change on trophic interactions. On average, experiments lasted 32.9 d (standard deviation [SD] ± 38.2), with a few experiments lasting over 100 d (*SI Appendix*, Fig. S2). Thus, the results are indicative of responses of marine organisms to relatively short-term acclimation. The environmental conditions used in the experiments were comparable to near-future scenarios of environmental change based on worst-case scenarios (mean ± SD values: temperature change, +3.808 ± 1.176 °C; partial pressure of CO_2_ (*p*CO_2_) change, +770.9 ± 563.0 µatm; or pH change, −0.374 ± 0.152). Finally, ∼51.3% of the studies manipulated both OA and warming in a fully factorial manner, and only 2.3% of all studies factorially manipulated consumer and resource exposure to any environmental-change variables.

**Fig. 2.**
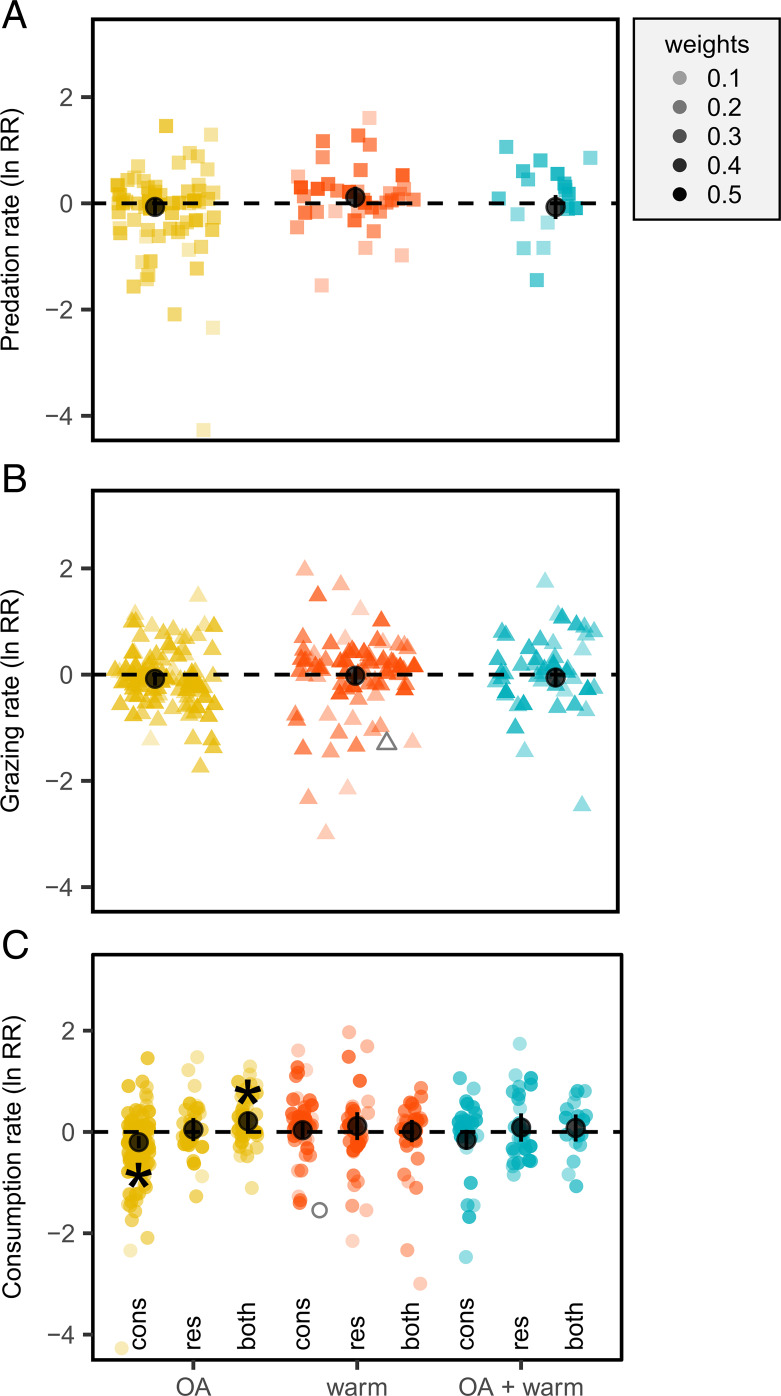
Individual and combined effects of OA and warming (warm) on consumption based on all studies combined (*A* and *B*) and by trophic role(s) exposed to future conditions (*C*). Points represent effect sizes (ln RR) per study, with opacity indicating their relative weight, overlaid with mean effect sizes (± 95% CI) on predation (*A*), grazing (*B*), and predation and grazing combined (*C*) among studies that exposed only consumers (cons), only resources (res), or both consumers and their resources (both) to experimental conditions (*C*). Unfilled gray points are outliers. *P < 0.05.

At the broadest scope, our meta-analysis highlights high variability in the effects of these near-future OA, warming, and combined OA and warming treatments on consumption rates (Fig. 2 *A* and *B*). This variability is consistent between studies focused on predation and herbivory (*SI Appendix*, Table S1), suggesting this result is not influenced by the type of consumption under consideration. As such, we did not detect an overall change in predation or herbivory for any environmental treatment when all studies were combined (Fig. 2 *A* and *B* and *SI Appendix*, Table S1). We emphasize the “high variability” over the “no overall effect” result, because there are numerous instances of statistically significant increases or decreases in consumption across all of the environmental-change variables analyzed here, suggesting these variables do affect consumption rates but not necessarily in a consistent way. However, the lack of an overall effect is not as simple as two very different populations of responses, such as increased and decreased consumption rates, cancelling each other out when synthesized together. Histograms of effect sizes for each environmental-change variable are qualitatively unimodal and centered near zero, with long tails skewed toward negative effect sizes representative of decreased consumption (*SI Appendix*, Fig. S3). This suggests that the analyses are not obscuring two very different populations of responses and the heterogeneity or structure that exists (QE_385_ = 3,988, *P* < 0.0001; *SI Appendix*, Table S1) is more nuanced. High variability in consumption in response to environmental change among marine organisms is in contrast to findings of previous meta-analyses (23) and suggests that we cannot assume that environmental change will uniformly increase or decrease consumption rates in the future, based on current empirical data.

**Fig. 3.**
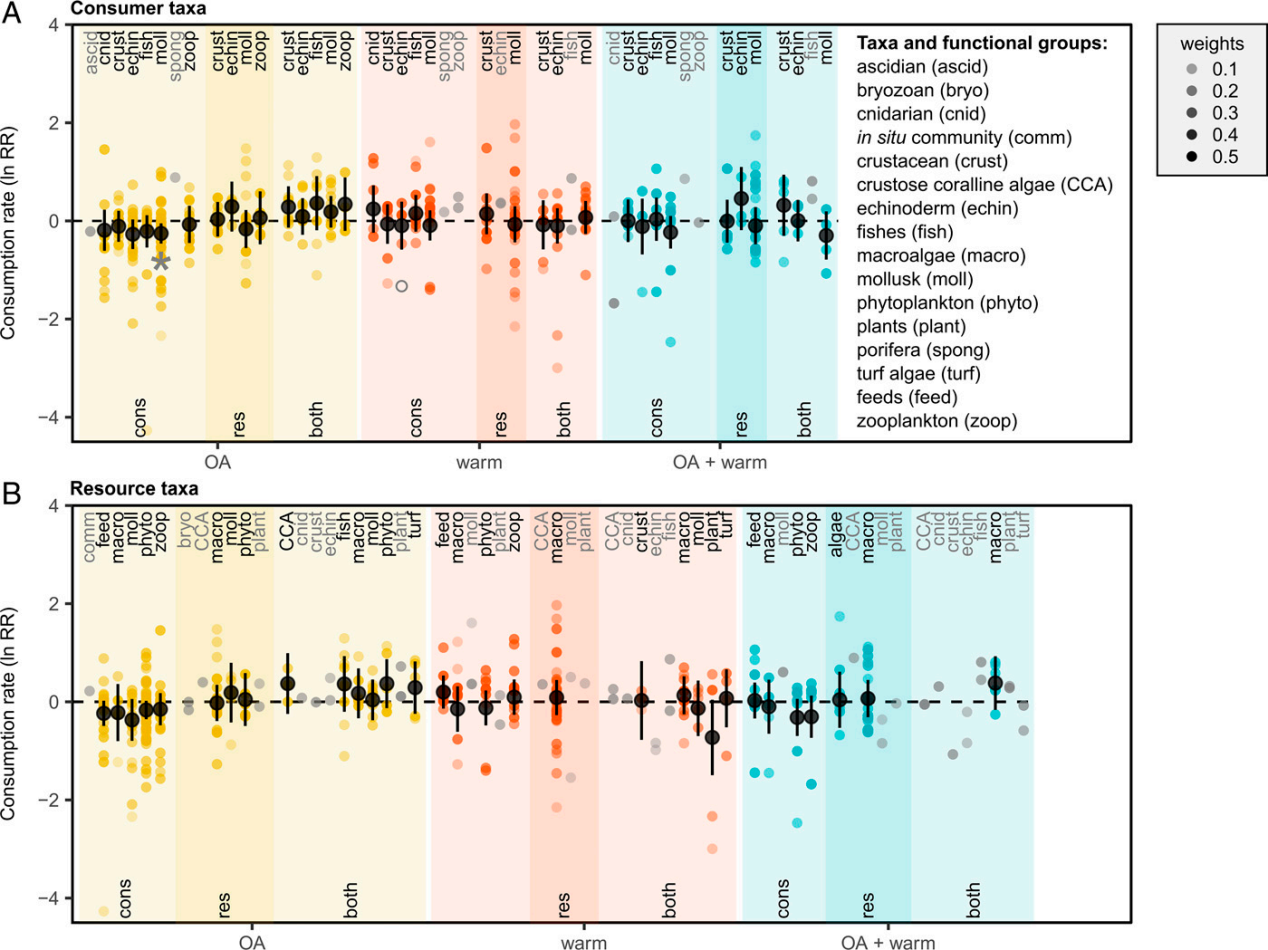
Individual and combined effects of OA and warming (warm) on consumption by trophic role and taxa or functional groups of consumers (cons) (*A*) and resources (res) (*B*). Points represent effect sizes (ln RR) per study, with opacity indicating their relative weight, overlaid with mean effect sizes (± 95% CI) on consumption among studies that exposed only consumers, only resources, or both consumers and their resources (both) to experimental conditions. Unfilled gray points are outliers and filled gray points are in groupings with k < 3 studies. A gray asterisk (*) indicates 95% confidence intervals that do not overlap 0 from models with moderators that were not significant.

Some of the variation in response, however, can be explained by methodological factors that can have important implications for our interpretation and inference in nature. For example, we demonstrate that the effects of some environmental-change factors differ depending on which trophic role (i.e., consumer or resource or both) was exposed to near-future conditions (Fig. 2*C* and *SI Appendix*, Table S1; note predation and herbivory were analyzed together in all subsequent analyses). In particular, we show that exposure to near-future OA tends to decrease consumption rates on average when only the consumer is exposed to these conditions. In contrast, when both the consumer and resource are exposed to near-future OA conditions, consumption rates actually increase on average (Fig. 2*C* and *SI Appendix*, Table S2). These patterns are not driven by particular taxa: all taxa showed similar trends in the consumer-only, resource-only, and consumer + resource experiments exposed to near-future OA (Fig. 3 and *SI Appendix*, Tables S1 and S2: treatment × trophic role × taxa *P* > 0.05). It is unclear why this disparity occurs, because there are few published studies that factorially manipulated consumers and resources (*n* = 4 articles) that can inform our interpretation, but we hypothesize that it could be driven by physiological or behavioral responses of the prey or resource that increases their vulnerability or palatability and thus overcompensates for a reduction in consumer performance. For example, a study of copepod consumption of phytoplankton at ambient and elevated *p*CO_2_ found that ingestion rates of the copepods did not differ among treatments when they were fed phytoplankton acclimated at ambient or low *p*CO_2_ conditions (i.e., a consumer-only experiment) (39). However, consumption increased when copepods acclimated to elevated *p*CO_2_ were given phytoplankton raised in the same conditions, which was attributed to a change in the fatty acid composition of phytoplankton in these conditions (39). Similarly, the pattern borne out in the present meta-analysis (i.e., reduced consumption in consumer-only treatments, but increased consumption in consumer + resource treatments) was reflected in a study of tropical reef-fish responses to OA. Here, both the predator and prey fishes demonstrated altered behaviors, but the increased predation in the consumer + resource treatments was attributed to reductions in escape performance (40). Thus, while studies that measure the effects of environmental change on consumption when only the consumer is exposed to these conditions can provide important mechanistic insight into consumer responses, they do not capture the potential emergent effects in nature when both consumers and their resources will be exposed to the same conditions.

In contrast to OA, the high variation in effect sizes in response to warming is consistent across studies that treated only the consumer, only the resource, or both (Fig. 2*C*), resulting in no overall effect of warming on consumption rates. Moreover, this pattern of high variation and no overall effect is repeated among different taxonomic groups of consumers or resources (Fig. 3 *A* and *B* and *SI Appendix*, Tables S1 and S2). This finding suggests that variation in response to warming is unlikely to be related to trophic role (as in ref. 30) or taxonomy (e.g., where some taxa are consistently more responsive to warming than others due to taxa-specific traits). Based on metabolic theory of ecology, we might expect warming to generally increase consumption when the magnitude of temperature change is not physiologically stressful (29–31). However, species’ responses to temperature are known to be nonlinear, with many marine species existing close to their thermal optimum (41). Metabolic theory has focused on the linear portion of species’ thermal performance curves, and it does not take into account how species might respond to warming that is beyond their thermal optimum or is physiologically stressful. In this case, we would expect reduced consumption as consumers surpass the thermal thresholds for optimal physiological performance (42–44). Thus, differences in how close current environmental temperatures are to a population’s thermal optimum and the degree of environmental change that populations will experience in the near future could drive a wide range of responses in ecological processes such as consumption (45), even among closely related organisms or organisms with similar traits (46–48). Given the relatively small range of temperature change used in the experiments, we hypothesize that the high variability in response to warming is more likely to be related to differences in how close current environmental conditions used in a given study were to the focal population’s thermal optima than to differences in the magnitude of the temperature change.

Similar to the result highlighting high variation and no overall effect in response to warming, we found high variability in the combined effect of OA and warming on consumption (Figs. 2 and 3 and *SI Appendix*, Tables S1 and S2). The analysis of the subset of studies that factorially manipulated these factors (k = 199 studies) resulted in an interactive effect of 0.5284 (95% confidence interval [CI]: −0.0383–1.0950), which is generally interpreted as an additive effect (i.e., the 95% CI for the interactive effect crosses zero). Although the interactive effect is nonsignificant, the trend is suggestive of a synergistic cumulative effect based on the individual effects of OA and warming nominally increasing consumption in this subset of studies, albeit nonsignificantly (*SI Appendix*, Table S3). Visual inspection of the patterns across all studies (Fig. 2*C*) suggests that any general effects of OA on consumption (e.g., decreased consumption when only consumers are exposed to OA versus increased consumption when both consumers and resources are exposed to OA) may be overridden by concurrent exposure to warmer temperatures, due to the high variability in response to warming.

### Gaps in Understanding: Future Research Directions.

Among the types of studies we examined, those that exposed both trophic roles to the combined effects of OA and warming provided the most accurate insight regarding how consumer-resource interactions will likely shift in future conditions, yet this study design was used the least (k = 17 studies from 12 published articles; Fig. 1). Although we have made progress in understanding various mechanisms by which individual environmental variables can directly and indirectly influence consumers or their resources, there remains a lag in our understanding of the mechanisms underlying combined effects (e.g., additive, synergistic) of these environmental variables on consumption. Furthermore, incorporating variability when simulating current and near-future local conditions would enhance our ability to infer future responses in natural systems from controlled experiments conducted in laboratory aquaria or mesocosms.

Although the overall effect of OA on consumption rates depended on which trophic roles were exposed to future conditions, only three published articles in our database (40, 49, 50) tested the mechanisms underlying changes in prey vulnerability in future conditions. A small number of studies (k = 8 studies) measured prey survival in future OA conditions by first exposing prey to treatment conditions in controlled laboratory settings and then quantifying prey survival in situ with the assumption that any loss of individuals was a result of mortality from natural predator communities. This distinction in experimental design impedes direct comparison with studies that quantified consumption rate, but analyses of these studies suggest future OA can increase prey vulnerability by 14.04% (95% CI: 5.43–31.74%; *SI Appendix*, Fig. S4 and Tables S1 and S2). We also note that our understanding of the effect of OA on prey survival is heavily informed by studies on tropical-reef fishes, and the results from this body of work have recently been challenged (51).

Prey responses can influence consumption rates a number of different ways, ranging from alterations in behavior to changes in size, palatability, or defensive structures (4). The only studies that assessed choice experiments with predators and prey (k = 2 studies) were excluded due to the predators being invasive species. Our analysis of choice experiments did not detect an effect of environmental-change drivers on choice of resource when herbivores in control conditions were offered resources maintained in control or environmental-change conditions (*SI Appendix*, Tables S1 and S2). There was, however, a trend toward a decrease in preference of resources that had been acclimated to warmer temperatures (*SI Appendix*, Fig. S5). Thus, we highlight the need for more studies that mechanistically test how prey or resource responses to global change mediate the outcomes of trophic interactions in future conditions.

Among the subset of studies that defined the life-history stages of study organisms (k = 231 studies), we noticed a trend whereby studies that solely quantified consumer responses typically focused on earlier life-history stages of consumers (*SI Appendix*, Fig. S6), which are predicted to be more vulnerable to OA and warming (1, 2). In contrast, studies that exposed both trophic roles to environmental change mostly examined later life-history stages of consumers; thus far, larval stages have been disregarded almost entirely (*SI Appendix*, Fig. S6). Direct comparisons between juvenile and adult life-history stages within a single species suggest that adults may be less likely to exhibit compensatory feeding in order to meet altered energetic demands when exposed to OA (52) or warming (53).

## Conclusions

The emergent effects of environmental change will be dependent on both consumer and resource responses, but our understanding of the mechanisms underlying this result is limited. Although environmental change can have clear, direct effects on the physiology, behavior, and energetic demands of consumers, the high variability in consumer responses to warming, in particular, limits general predictions for the future ocean. We argue that this variability in response to warming is consistent with predictions based on nonlinear thermal performance curves and variation in local adaptation to the thermal environment among species (45, 50). Moreover, when species are exposed to OA and warming in combination, the effects are generally additive and reflect the high variability that is demonstrated in response to warming. Although some of the variation in response (at least for OA) is related to consumer versus resource responses, as well as life stages of taxa, there are likely other sources of variation that have not been captured in this analysis (e.g., ref. 45). Importantly, our results highlight the challenges inherent in directly translating organismal response to environmental variables into accurate predictions of future trophic interactions—and ultimately, community structure—based on the current empirical evidence.

As the need for accurate predictions of the future state of natural systems grows increasingly urgent, meta-analysis will continue to serve as a critical tool to ascertain general patterns in ecological response. It is critical, however, to remember that the choice of which studies to include in meta-analyses can profoundly influence the results (54, 55). We have shown that conclusions may be misleading when synthesizing across broadly measured metrics of response [e.g., consumption rate (23)] without considering underlying sources of variance among studies, such as differing experimental designs (e.g., which trophic roles were exposed to future conditions) and patterns in the taxa and life-history stages examined. It is also important to note that meta-analysis can provide new hypotheses for laboratory and field studies. By highlighting patterns or gaps that emerge from a broader body of literature, synthesis can feed back into empirical work and push the field forward. As such, we have identified the need for more research on the combined effects of multiple environmental variables, the resource-driven component of trophic response, and a broader range of pairwise combinations of taxa and species’ traits. By highlighting these gaps, research efforts can be strategically leveraged to minimize the number of studies required to better understand any environmental control on higher-level ecological responses.

## Materials and Methods

### Systematic Review.

We searched for relevant articles published up to July 27, 2019 that reported the effects of OA and/or warming on per capita consumption rates of predator–prey and herbivore–resource interactions. In the Web of Science database, we searched for keywords of environmental conditions paired in every combination with keywords of biological parameters, with the additional terms “ocean” or “marine” as needed (see *SI Appendix*, Fig. S1 and Dataset S1 for Boolean search terms). We removed duplicate records and then imported the 38,019 titles and abstracts into the semiautomated screening tool, Abstrackr (56). This tool greatly reduces screening burden (57, 58); machine-learning algorithms order the presentation of abstracts for review based on the probability of meeting inclusion criteria. We reviewed a total of 7,665 abstracts for relevancy, at which point the algorithms deemed all remaining abstracts to be exclusion worthy, given how unlikely they were to be appropriate. We were able to retrieve 494 full texts of the articles that seemed potentially relevant, from which we found 131 articles with suitable data (*SI Appendix*, Fig. S1). We also included eight additional prescreened articles that were found during a preliminary search using Google Scholar that were not included in the search results in Web of Science.

We considered any studies reported within each article that measured consumer–resource interactions in clearly defined conditions of both present day and projected changes in the carbon chemistry and/or temperature of seawater predicted for the year 2100 based on the “worst-case” emmission scenario defined by the Intergovernmental Panel on Climate Change (59). This decision was based on the vast majority of studies using environmental conditions associated with this scenario. Control conditions in studies needed to represent the current, ambient conditions of each study system, which were typically based on previously conducted or ongoing measurements of field conditions. We also did not consider studies if herbivores were fed paint or if interactions involved non-native organisms.

### Data Extraction and Calculation of Effect Sizes.

We mined data from our final database of 434 studies from 133 published articles, using the software program Data Thief III (version 1.7; https://www.datathief.org/) and contacting authors directly as needed to obtain all necessary data. All studies in our final database that simulated OA either raised *p*CO_2_ (+770.9 ± 563.0 µatm [mean ± SD]) or lowered pH (−0.374 ± 0.152 [mean ± SD]) by bubbling the experimental seawater with CO_2_-enriched gas. We also included studies from three articles in which acid additions were performed without accompanying additions of bicarbonate (HCO_3_) to reduce the pH of seawater (−0.390 ± 0.090 [mean ± SD]), after verifying from preliminary sensitivity analyses that their exclusion did not alter the overall results. Warming treatments increased temperature by 3.808 ± 1.176 °C (mean ± SD). If additional environmental variables were also manipulated (e.g., nutrients, salinity), we included responses only in the ambient levels of these factors.

We extracted per capita consumption rates of carnivores and herbivores, as well as proportional metrics of prey survival. In instances of multiple measurements of response per individual repeated through time, we included the response from only the final time point. We also extracted information from each study indicating whether consumers and/or resources were exposed to treatment conditions, or if treated and untreated resources were concurrently available to a single consumer when quantifying consumption rate (hereafter referred to as “choice experiments”). The only choice experiments of predation involved non-native prey and thus were not further assessed. All remaining choice experiments measured grazing rates of an herbivore maintained in ambient conditions, with the exception of only two studies (which were excluded due to this low sample size) that exposed both herbivores and their resources to treatment conditions. From all studies, we identified the taxa of predators, prey, and herbivores, and functional groups of autotrophs. We also extracted the life-history stages of predators, prey, and herbivores from a subset of studies that provided such information about the study organisms.

All data manipulation and analyses were conducted using the statistical software, *R* (version 3.6.2) (60), with the associated packages *tidyverse* (version 1.3.0) (61) and *metafor* (version 2.4–0) (62). We calculated the log-transformed response ratio of means (ln[RR] = ln[Response Future/Response Ambient]) (63) of the individual and combined effects of OA and warming on consumption rates measured in each study using the *escalc* function from the *metafor* package, whereby positive values indicate higher consumption rates in future versus ambient conditions. Among choice studies, this ratio is the relative grazing on concurrently available resources exposed to future versus ambient conditions, with positive values indicating enhanced grazing (i.e., preference) on future resources. Due to the chosen metric of ln(RR), we were unable to calculate effect sizes for observations with responses of zero (*n* = 4 studies) or responses that differed in signs between ambient and future treatments (*n* = 5 studies). We also converted reported binomial proportions of prey survival into 2 × 2 tables of counts of prey that did versus did not survive in future versus ambient conditions to calculate log-transformed odds ratios of response: ln(odds_surv_future_/odds_surv_ambient_).

### Data Diagnostics.

For each response metric (i.e., consumption rate, prey survival, and grazing rate among choice experiments), we fit a series of three-level meta-analysis models with restricted maximum-likelihood estimation (REML) using the *rma.mv* function in the *metafor* package and included effect size nested within publication as random effects to account for nonindependence of multiple effect sizes from individual studies. We first fit models per response variable and assessed outlier and influential observations based on Cook’s distance, DFBETAS, studentized residuals, and diagonal values along the hat matrix (*SI Appendix*, Fig. S7), as well as sensitivity analyses (*SI Appendix*, Table S4) that indicated removal of apparent outliers from models would result in more conservative effect-size estimates. All outliers were removed from models prior to subsequent analyses. We conducted Egger's regression tests to assess publication bias, whereby the standard errors of effect size estimates were included as a moderator in models and the significance of the corresponding coefficient indicates the symmetry (“significant” indicates asymmetric distribution; *SI Appendix*, Table S4). Results from these tests provided no indication of asymmetry in any of the models.

### Analysis.

To test the effects of OA and warming on consumption rates and whether response varies by the type of interaction (predation or grazing), we fit a series of three-level meta-analysis models with REML (64–66) using the *rma.mv* function in the *metafor* package, with effect size nested within publication as random effects to account for between- and within-publication heterogeneity and sampling variability. Each model included one of the following moderators: (1) environmental variable tested (treatment: OA, warming, OA + warming); (2) consumption type (predation vs. grazing); or (3) the interaction between the two variables (treatment × consumption type). Effect-size weightings consisted of the inverse of the variance–covariance matrix implied by each model (i.e., weight matrix rather than a diagonal matrix given the multilevel and multivariate data). After finding no indication of consumption type being a significant moderator, we did not continue testing this variable in subsequent models.

Next, we tested whether response varied according to which role of the trophic interaction (trophic role: consumer-only, resource-only, or consumer–resource) was exposed to future conditions (moderators: treatment + trophic role + treatment × trophic role). To test for additional sources of variation in consumption rate, subsequent models were fit as described in the preceding paragraph but with three-way interaction terms as moderators that consisted of treatment × trophic role in combination with one of the following variables: (1) consumer taxa, (2) resource taxa (prey) or functional group (resource), (3) consumer life-history stage, and (4) prey life-history stage. To test the effects of OA and warming on relative grazing on future versus ambient resources measured from choice experiments, we fit a mixed-effects model but only had enough studies to test the significance of the treatment moderator. We did not include any moderators in a model of prey survival, due to all studies having exposed only prey to OA treatments and a low number of studies that prevented further investigation of additional sources of variance in response.

Although the Knapp and Hartung adjustment, which helps account for the uncertainty in the estimate of residual heterogeneity (67), cannot be directly applied to multilevel models, we were able to apply a similar approach to assess the significance of moderators in the models. We used Omnibus tests based on an F distribution and used a *t* distribution for tests of model coefficients, which resulted in more conservative estimates. We also compared the corresponding 95% CIs of coefficients with bootstrapped 95% CIs based on 10,000 iterations that were calculated using the *boot* package (version 1.3–24) (68, 69); we found the nonbootstrapped values tended to result in wider CIs and fewer instances of CIs that did not overlap zero (*SI Appendix*, Tables S2 and S3). We therefore report in the figures and main text only the 95% CIs estimated with a *t* distribution. We assessed the heterogeneity of response in every model by calculating a variety of statistics (70) and report estimates of residual heterogeneity (QE), between-publication variance (σ^2^_between_, equivalent to τ^2^), within-publication variance (σ^2^_within_), and the distribution of variance (relative percent) across levels (*I*^2^_between_ and *I*^2^_within_).

Finally, we calculated the interactive effect of OA and warming for the subset of studies that factorially manipulated temperature and carbonate chemistry. Rather than using means and SDs calculated from the raw responses reported by each study, we calculated weighted inputs by fitting a three-level meta-analysis model of treatment response (control, OA, warming, OA and warming) with REML, effect size nested within publication as random effects, and treatment as a moderator. We then used the weighted estimates and SEs per treatment from the model to calculate the overall interactive effect, using the formulas outlined by Morris et al. (71).

## Supplementary Material

Supplementary File

Supplementary File

Supplementary File

Supplementary File

Supplementary File

Supplementary File

Supplementary File

## Data Availability

We have provided the database of previously published data, datasets used for analyses, and code that reproduces our analysis as Datasets S2–S6.
