# Supplementary material for: Emergent effects of global change on consumption depend on consumers and their resources in marine systems
Source: Proc Natl Acad Sci U S A. 2022 Apr 21;119(18):e2108878119. doi: 10.1073/pnas.2108878119 (PMC9173678; doi:10.1073/pnas.2108878119)
Supplement: Supplementary File [file pnas.2108878119.sapp.pdf]

PNAS
www.pnas.org

Supplementary Information for

Emergent effects of environmental change on consumption depend on consumers and their resources in marine systems

Tye L. Kindinger, Jason A. Toy, and Kristy J. Kroeker

Kristy J. Kroeker
Email: kkroeker@ucsc.edu

This PDF file includes:

Figures S1 to S7
Tables S1 to S4
Legends for Datasets S1-S5
SI References

Other supplementary materials for this manuscript include the following:

Dataset S1
Dataset S2
Dataset S3
Dataset S4
Dataset S5
Dataset S6

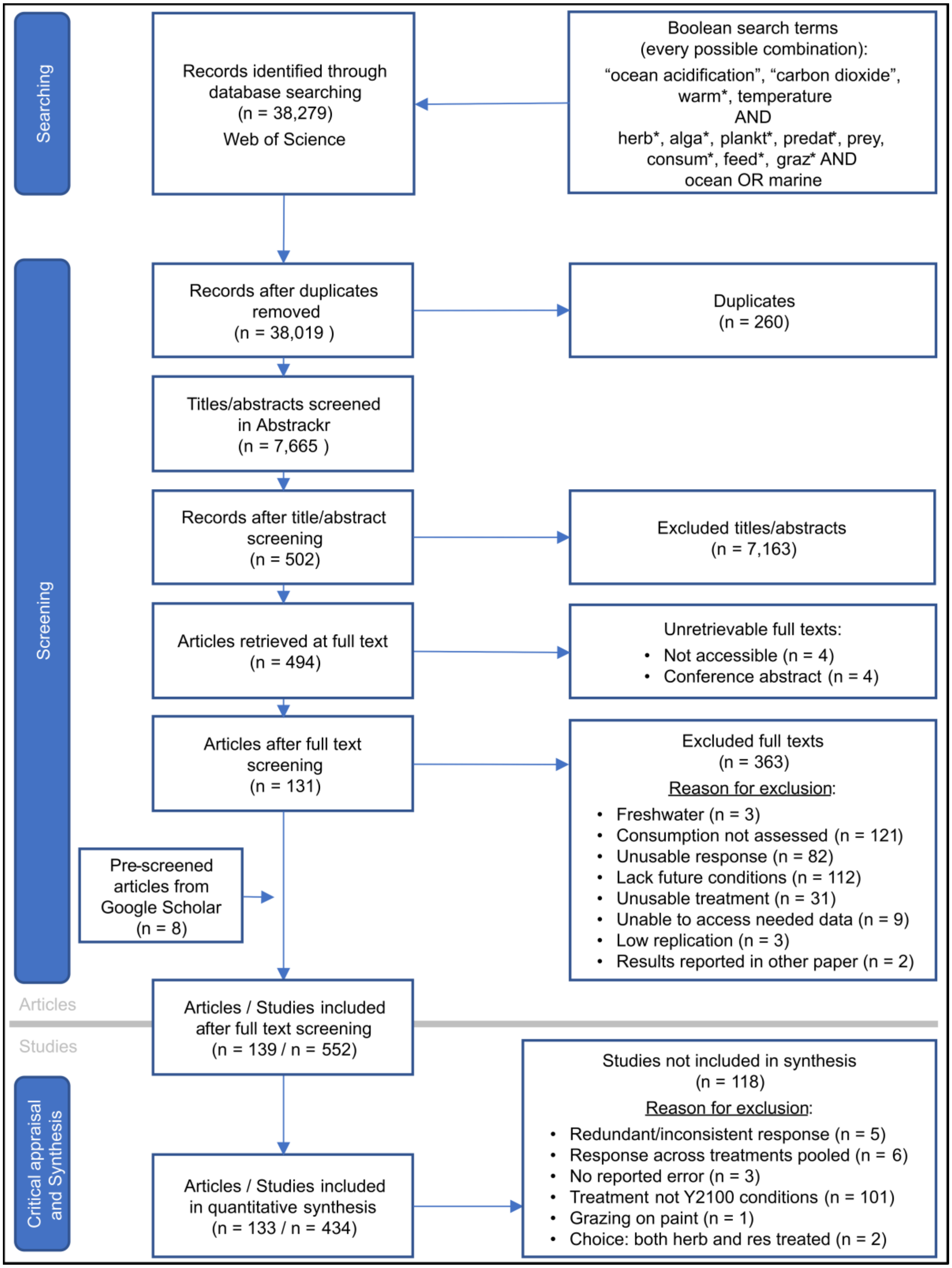

Fig. S1. Overview of systematic-review procedure. Modified flow diagram from RepOrtingstandards for Systematic Evidence Syntheses [ROSES (1)].

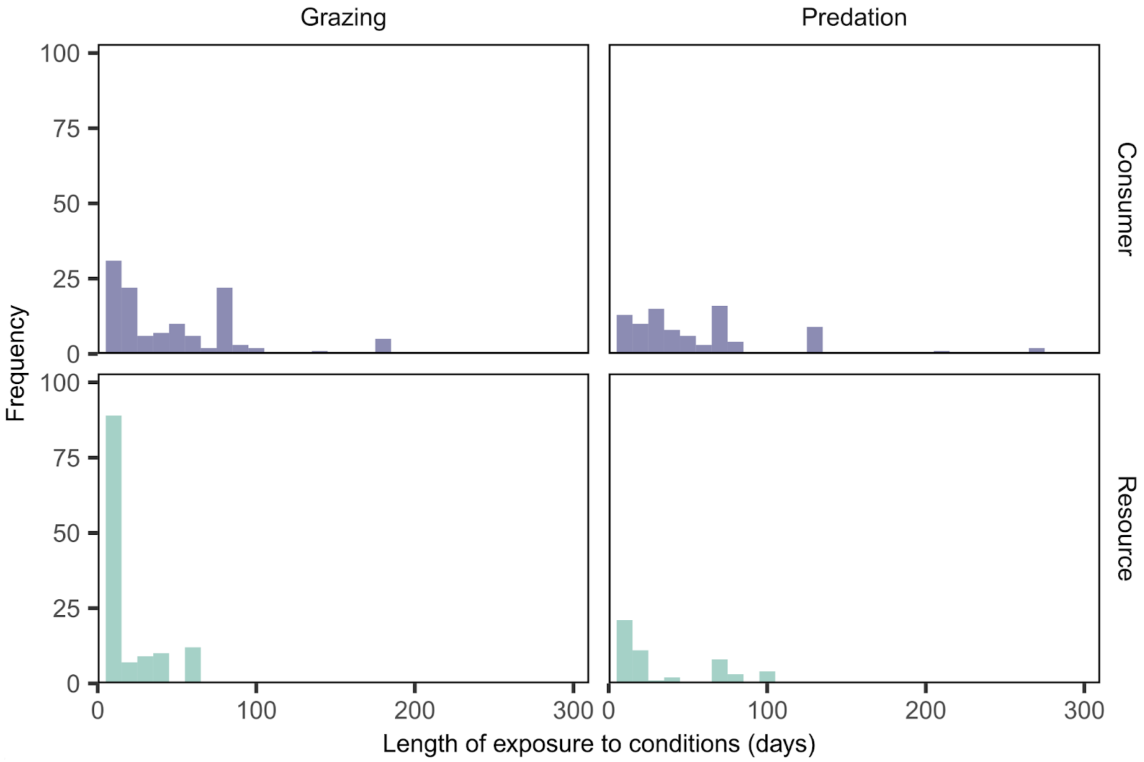

Fig. S2. Frequency of exposure length (days) of consumers and resources to experimental conditions among studies that measured grazing or predation rates.

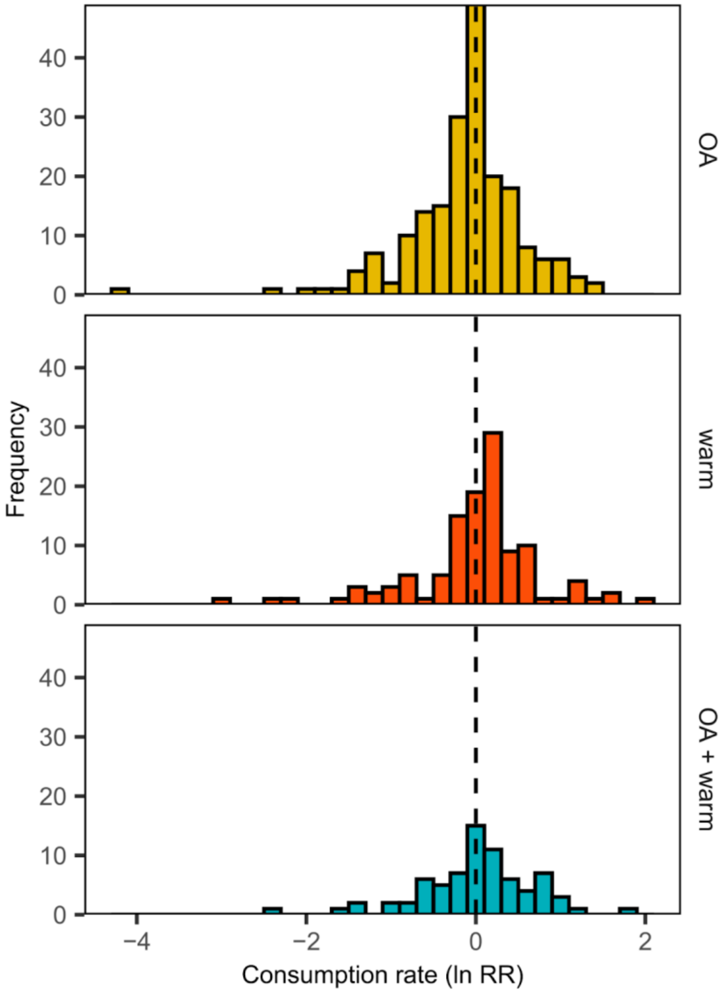

Fig. S3. Frequency of effect sizes (ln RR) per study of the individual and combined effects of ocean acidification (OA) and warming (warm) on consumption rate.

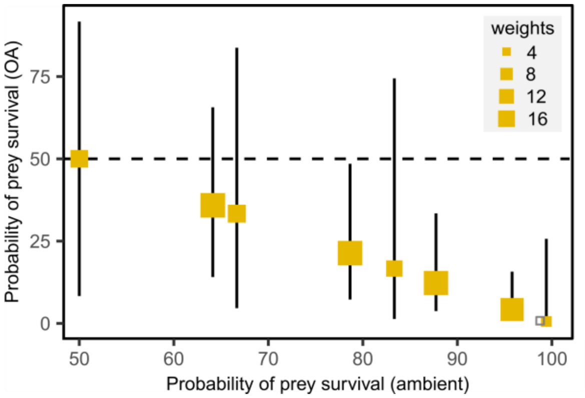

Fig. S4. Probability of prey survival among experiments that exposed prey to future ocean acidification (OA) or ambient conditions in the lab and then quantified survival in situ with assumed mortality from natural predator communities. Points below the dashed line are studies with a probability of OA-treated prey survival less than 50% (± 95% CI) and the unfilled grey point is an outlier. The size of points represents the relative weight of effect sizes (log odds ratio) in the corresponding model, which estimated an overall mean of relative survival in OA versus ambient conditions of -1.8119 (95% CI: -2.8581 to -0.7658).

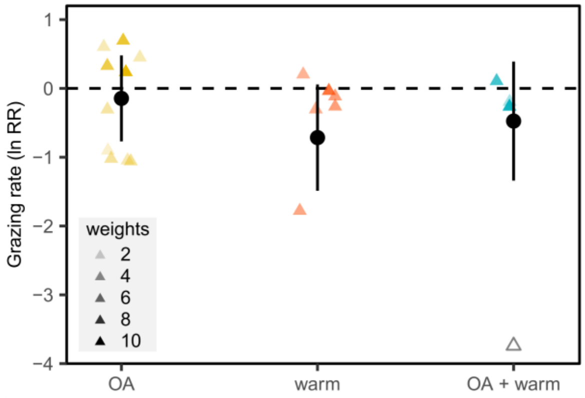

Fig. S5. Individual and combined effects of ocean acidification (OA) and warming (warm) on grazing among "choice" experiments whereby treated and untreated resources were concurrentlyavailable to a single consumer. Points represent effect sizes (ln RR) per study consisting of relative grazing on resources exposed to treatment versus ambient conditions, overlaid with mean effect sizes (± 95% CI). Positive values indicate greater rates of grazing on treated resources and negative values indicate greater rates of grazing on control resources. The unfilled grey point is an outlier and the opacity of points indicate the relative weight of effect sizes in the corresponding model.

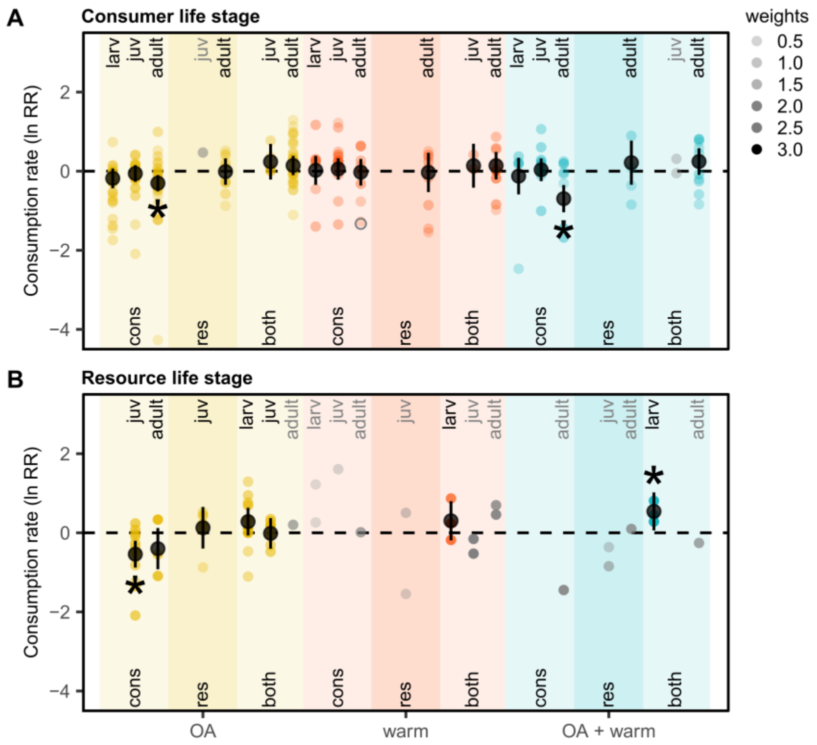

Fig. S6. Individual and combined effects of ocean acidification (OA) and warming (warm) on consumption rates of larval (larv), juvenile (juv) and adult life-history stages of consumers (*A*) and resources (*B*). Points represent effect sizes (ln RR) per study with opacity indicating their relative weight, overlaid with mean effect sizes (± 95% CI) among studies that exposed consumers (cons), resources (res) or both to experimental conditions. The unfilled grey point is an outlier, and filled grey points are in groupings with k < 3 studies. An asterisk (*) denotes a significant effect (P<0.05).

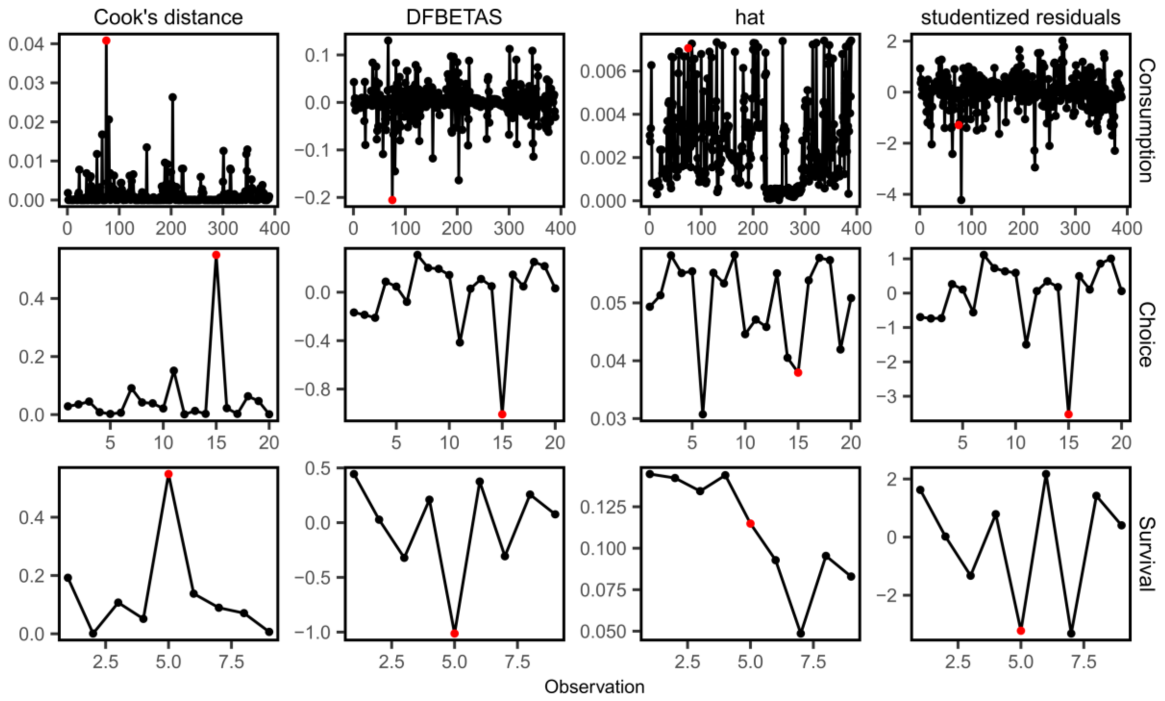

Fig. S7. Outlier and influential case diagnostics per response metric (consumption rate, grazing rate from choice experiments, and prey survival). Values were calculated from three-level meta-analysis models fit without moderators using restricted maximum-likelihood estimation (REML) and included Effect Size nested within Publication as random effects. Red points were observations identified as outliers with Cook's distance values greater than 4/k studies.

Table S1. Summary statistics of Omnibus tests of moderators and heterogeneity from models of consumption, grazing across choice experiments, and prey survival. Moderators tested were *Consumption Type* (predation or grazing) and the environmental variables, ocean acidification and/or warming (*Treatment*), as well as environmental variables in combination with (a) which trophic roles of an interaction were exposed to environmental-variable treatments (*Trophic Role*); (b) *Taxa* and (c) *Life Stage* of consumers (cons) or resources (res) observed; and (d) whether herbivores were exposed concurrently to treated and untreated resources in choice experiments. Moderators with significant p-values (p<0.05) are in bold. Corresponding figures per model are listed.

				Omnibus test				Heterogeneity					
Resp	Moderator	Fig	k	F	df_m	df_{k-p}	p-value	$QE_{df(k-p)}$	$QE_{p\text{-}value}$	$\sigma^2_{(btw)}$	$\sigma^2_{(w/in)}$	$I^2_{(btw)}$	$I^2_{(w/in)}$
Consumption	Consumption Type × Treatment	2A,B	388	0.8240	5	382	0.5331	3780.1	<0.0001	0.1509	0.1231	54.354	44.330
	Consumption Type + Treatment		388	0.9567	3	384	0.4132	3972.7	<0.0001	0.1517	0.1219	54.710	43.972
	Consumption Type		388	0.2357	1	386	0.6276	4936.1	<0.0001	0.1531	0.1229	54.752	43.941
	Treatment		388	1.3164	2	385	0.2693	3988.0	<0.0001	0.1505	0.1219	54.518	44.158
	**Treatment × Trophic Role**	2C	388	2.5563	8	379	**0.0101**	3589.6	<0.0001	0.1319	0.1223	51.160	47.422
	Treatment × Trophic Role × $Taxa_{cons}$	3A	388	1.2842	44	343	0.1157	2661.8	<0.0001	0.1504	0.1156	55.779	42.866
	Treatment × Trophic Role × $Taxa_{res}$	3B	388	0.8983	58	329	0.6836	2887.0	<0.0001	0.1587	0.1286	54.547	44.196
	**Treatment × Trophic Role × $\textbf{Life Stage}_{\textbf{cons}}$**	S6A	225	2.0546	18	206	**0.0086**	1356.9	<0.0001	0.1747	0.0692	70.740	28.007
	**Treatment × Trophic Role × $\textbf{Life Stage}_{\textbf{res}}$**	S6B	64	2.6997	17	46	**0.0038**	130.55	<0.0001	0.0245	0.1109	14.2769	64.659
Grazing	Treatment (choice)	S5	19	1.7262	2	16	0.2095	121.77	<0.0001	0.3752	0.1197	68.1660	21.748
**Prey survival**	*NA* (all OA)	S4	8	14.473	1	7	**0.0067**	12.6025	0.0824	<0.0001	0.5167	<0.0001	40.823

Table S2. Summary statistics from models of the effects of ocean acidification (OA), warming (W), or both (OW) in combination with additional moderators on consumption. 95% confidence intervals were compared with those calculated by bootstrapping over 10,000 iterations (boot). Levels with significant p-values (p<0.05) and/or 95% CIs zero are in bold.

Moderator	Moderator level	k	Coefficients				95% CI		95% CI (boot)	
			estimate	SE	t-value	p-value	lower	upper	lower	upper
Consumption Type × Treatment	herb.OA	118	-0.0780	0.0660	-1.1823	0.2378	-0.2077	0.0517	-0.1222	0.0292
	herb.OAW	51	-0.0550	0.0904	-0.6081	0.5435	-0.2326	0.1227	-0.0944	0.1564
	herb.W	78	-0.0213	0.0788	-0.2699	0.7874	-0.1761	0.1336	-0.1582	0.0942
	pred.OA	81	-0.0673	0.0832	-0.8091	0.4190	-0.2308	0.0962	-0.1127	0.0861
	pred.OAW	23	-0.0697	0.1146	-0.6085	0.5432	-0.2950	0.1556	-0.2895	0.2270
	pred.W	37	0.1153	0.1008	1.1439	0.2534	-0.0829	0.3134	**0.0140**	**0.2795**
Treatment × Trophic Role	**OA.both**	**46**	**0.2117**	**0.0947**	**2.2354**	**0.0260**	**0.0255**	**0.3979**	**0.1534**	**0.3467**
	**OA.cons**	**117**	**-0.2017**	**0.0608**	**-3.3141**	**0.0010**	**-0.3213**	**-0.0820**	**-0.2750**	**-0.1161**
	OA.res	36	0.0469	0.1162	0.4035	0.6868	-0.1816	0.2753	-0.0658	0.1538
	OAW.both	17	0.0779	0.1364	0.5714	0.5681	-0.1903	0.3462	-0.1559	0.2642
	OAW.cons	29	-0.1560	0.0979	-1.5941	0.1117	-0.3485	0.0364	-0.3975	0.0897
	OAW.res	28	0.0873	0.1424	0.6135	0.5399	-0.1926	0.3672	-0.1329	0.2396
	W.both	35	0.0164	0.1121	0.1465	0.8836	-0.2040	0.2369	-0.1237	0.2000
	W.cons	46	0.0348	0.0851	0.4087	0.6830	-0.1325	0.2020	-0.0696	0.2097
	W.res	34	0.1157	0.1399	0.8269	0.4088	-0.1594	0.3907	-0.1348	0.2468
Treatment × Trophic Role × $Taxa_{cons}$	OA.both.crust	9	0.2840	0.2144	1.3249	0.1861	-0.1376	0.7057	**0.0377**	**0.4416**
	OA.both.echin	10	0.0902	0.1900	0.4749	0.6352	-0.2835	0.4640	-0.0313	0.2305
	OA.both.fish	11	0.3612	0.2808	1.2863	0.1992	-0.1911	0.9135	**0.0273**	**0.5527**
	OA.both.moll	12	0.1861	0.1640	1.1350	0.2572	-0.1364	0.5087	**0.1102**	**0.4361**
	OA.both.zoop	4	0.3405	0.2768	1.2302	0.2194	-0.2039	0.8849	-0.1191	0.7451
	OA.cons.ascid	*1*	*-0.8719*	*0.4682*	*-1.8621*	*0.0634*	*-1.7928*	*0.0491*	*NA*	*NA*
	OA.cons.cnid	10	-0.1899	0.2131	-0.8912	0.3735	-0.6090	0.2292	-0.4760	0.4668
	OA.cons.crust	15	-0.1100	0.1608	-0.6842	0.4943	-0.4264	0.2063	-0.1535	0.0616
	OA.cons.echin	18	-0.2712	0.1537	-1.7646	0.0785	-0.5735	0.0311	**-0.5432**	**-0.0559**
	OA.cons.fish	13	-0.2127	0.1678	-1.2674	0.2059	-0.5427	0.1174	-0.5280	0.2607
	**OA.cons.moll**	**45**	**-0.2548**	**0.1047**	**-2.4321**	**0.0155**	**-0.4608**	**-0.0487**	**-0.3961**	**-0.1341**
	OA.cons.spong	*1*	*0.8841*	*0.6541*	*1.3517*	*0.1774*	*-0.4024*	*2.1706*	*NA*	*NA*
	OA.cons.zoop	14	-0.0711	0.1930	-0.3681	0.7130	-0.4507	0.3086	-0.3016	0.0048
	OA.res.crust	11	0.0333	0.1791	0.1860	0.8526	-0.3190	0.3857	-0.2661	0.2320
	OA.res.echin	5	0.2913	0.2590	1.1247	0.2615	-0.2181	0.8007	-0.0889	0.3653
	OA.res.moll	15	-0.1651	0.1972	-0.8373	0.4030	-0.5529	0.2227	-0.2753	0.0883
	OA.res.zoop	5	0.0625	0.2745	0.2276	0.8201	-0.4775	0.6025	-0.2528	0.1617
	OAW.both.crust	5	0.3193	0.3167	1.0084	0.3140	-0.3036	0.9422	-0.0570	0.7900
	OAW.both.echin	6	0.0048	0.2157	0.0221	0.9823	-0.4194	0.4290	-0.0243	0.2863
	OAW.both.fish	*2*	*0.7620*	*0.3721*	*2.0481*	*0.0413*	*0.0302*	*1.4938*	*NA*	*NA*
	OAW.both.moll	4	-0.2974	0.2498	-1.1905	0.2347	-0.7888	0.1940	-0.8824	0.1724
	OAW.cons.cnid	*2*	*-1.1758*	*0.3343*	*-3.5169*	*0.0005*	*-1.8334*	*-0.5182*	*NA*	*NA*
	OAW.cons.crust	7	0.0024	0.2213	0.0106	0.9915	-0.4330	0.4377	**0.1221**	**0.2968**
	OAW.cons.echin	3	-0.1146	0.2893	-0.3962	0.6922	-0.6836	0.4544	-0.7539	0.5667
	OAW.cons.fish	5	0.0341	0.2245	0.1518	0.8795	-0.4074	0.4755	-0.6152	0.6694
	OAW.cons.moll	10	-0.2316	0.1693	-1.3676	0.1723	-0.5647	0.1015	-0.7523	0.1059
	OAW.cons.spong	*1*	*0.8566*	*0.6650*	*1.2882*	*0.1985*	*-0.4513*	*2.1646*	*NA*	*NA*
	OAW.cons.zoop	*1*	*-0.0248*	*0.4992*	*-0.0496*	*0.9605*	*-1.0066*	*0.9571*	*NA*	*NA*
	OAW.res.crust	6	-0.0054	0.2230	-0.0241	0.9808	-0.4440	0.4333	-0.4190	0.2738
	OAW.res.echin	3	0.4556	0.3276	1.3909	0.1652	-0.1887	1.0999	**0.0162**	**0.6785**
	OAW.res.moll	19	-0.0968	0.1934	-0.5006	0.6170	-0.4773	0.2837	-0.3450	0.3190
	W.both.crust	8	-0.0796	0.2546	-0.3126	0.7548	-0.5805	0.4213	-0.1454	0.2611
	W.both.echin	11	-0.1016	0.1834	-0.5538	0.5801	-0.4622	0.2591	-0.4104	0.2554
	W.both.fish	*2*	*0.4088*	*0.3902*	*1.0477*	*0.2955*	*-0.3587*	*1.1763*	*NA*	*NA*
	W.both.moll	14	0.0702	0.1716	0.4089	0.6829	-0.2673	0.4076	-0.3011	0.2661
	W.cons.cnid	5	0.2425	0.2455	0.9878	0.3239	-0.2404	0.7255	-0.1117	0.9044
	W.cons.crust	8	-0.0634	0.2054	-0.3086	0.7578	-0.4673	0.3406	-0.5860	0.1953
	W.cons.echin	4	-0.0960	0.2486	-0.3861	0.6996	-0.5851	0.3930	-0.2017	0.3044
	W.cons.fish	11	0.1542	0.1904	0.8098	0.4186	-0.2204	0.5288	-0.0642	0.3743
	W.cons.moll	15	-0.0924	0.1566	-0.5902	0.5554	-0.4005	0.2156	-0.3615	0.2090
	W.cons.spong	*1*	*0.1796*	*0.6669*	*0.2693*	*0.7879*	*-1.1321*	*1.4913*	*NA*	*NA*
	W.cons.zoop	*2*	*0.3588*	*0.3784*	*0.9482*	*0.3437*	*-0.3855*	*1.1031*	*NA*	*NA*
	W.res.crust	8	0.1440	0.2122	0.6789	0.4976	-0.2733	0.5613	-0.2321	0.4960
	W.res.echin	*2*	*0.2840*	*0.4407*	*0.6445*	*0.5197*	*-0.5828*	*1.1507*	*NA*	*NA*
	W.res.moll	24	-0.0701	0.1866	-0.3756	0.7074	-0.4372	0.2970	-0.2844	0.2600

Treatment × Trophic Role × $Taxa_{res}$	OA.both.CCA	3	0.3723	0.3149	1.1822	0.2380	-0.2472	0.9918	**0.1356**	**0.4360**
	OA.both.cnid	1	0.0795	0.6359	0.1250	0.9006	-1.1715	1.3304	NA	NA
	OA.both.crust	1	-0.0267	0.5366	-0.0498	0.9603	-1.0823	1.0288	NA	NA
	OA.both.echin	2	0.1892	0.5620	0.3367	0.7366	-0.9164	1.2949	NA	NA
	OA.both.fish	11	0.3638	0.2884	1.2613	0.2081	-0.2036	0.9313	**0.0239**	**0.5547**
	OA.both.macro	6	0.1727	0.2584	0.6684	0.5044	-0.3357	0.6811	-0.2039	0.1648
	OA.both.moll	10	0.0389	0.2115	0.1841	0.8540	-0.3771	0.4549	**0.0993**	**0.2549**
	OA.both.phyto	5	0.3703	0.2536	1.4604	0.1451	-0.1285	0.8691	**0.1247**	**0.9152**
	OA.both.plant	2	-0.3244	0.4555	-0.7121	0.4769	-1.2204	0.5717	NA	NA
	OA.both.turf	5	0.2889	0.2714	1.0647	0.2878	-0.2449	0.8228	**0.1701**	**0.8578**
	OA.cons.comm	1	0.2198	0.5598	0.3926	0.6948	-0.8815	1.3210	NA	NA
	OA.cons.feed	22	-0.2350	0.1309	-1.7945	0.0737	-0.4926	0.0226	**-0.3791**	**-0.0064**
	OA.cons.macro	5	-0.2202	0.2962	-0.7434	0.4578	-0.8028	0.3624	-0.2839	0.1170
	OA.cons.moll	15	-0.3693	0.2170	-1.7019	0.0897	-0.7961	0.0576	**-0.8975**	**-0.1276**
	OA.cons.phyto	57	-0.1670	0.0955	-1.7489	0.0812	-0.3549	0.0208	**-0.3260**	**-0.1134**
	OA.cons.zoop	17	-0.1503	0.1669	-0.9005	0.3685	-0.4786	0.1780	-0.2577	0.2568
	OA.res.bryo	2	-0.0799	0.4969	-0.1607	0.8724	-1.0573	0.8976	NA	NA
	OA.res.CCA	1	0.3978	0.6233	0.6382	0.5238	-0.8284	1.6240	NA	NA
	OA.res.macro	20	-0.0230	0.1883	-0.1221	0.9029	-0.3934	0.3474	-0.1872	0.2339
	OA.res.moll	6	0.1872	0.3093	0.6051	0.5456	-0.4213	0.7957	**0.0701**	**0.4361**
	OA.res.phyto	5	0.0460	0.2743	0.1679	0.8668	-0.4936	0.5857	-0.2514	0.1621
	OA.res.plant	2	0.0749	0.4522	0.1655	0.8686	-0.8146	0.9643	NA	NA
	OAW.both.CCA	2	-0.0158	0.3904	-0.0404	0.9678	-0.7838	0.7523	NA	NA
	OAW.both.cnid	1	0.3114	0.5584	0.5577	0.5775	-0.7870	1.4098	NA	NA
	OAW.both.crust	1	-1.0897	0.5398	-2.0187	0.0443	-2.1517	-0.0278	NA	NA
	OAW.both.echin	2	-0.5050	0.6170	-0.8184	0.4137	-1.7189	0.7089	NA	NA
	OAW.both.fish	2	0.7593	0.3849	1.9726	0.0494	0.0021	1.5165	NA	NA
	OAW.both.macro	5	0.3832	0.2781	1.3781	0.1691	-0.1638	0.9302	-0.1375	0.4858
	OAW.both.plant	2	-0.3659	0.4596	-0.7961	0.4266	-1.2699	0.5382	NA	NA
	OAW.both.turf	2	-0.2189	0.4218	-0.5190	0.6041	-1.0487	0.6108	NA	NA
	OAW.cons.feed	9	0.0265	0.1866	0.1422	0.8870	-0.3406	0.3937	-0.2725	0.4860
	OAW.cons.macro	5	-0.0994	0.2804	-0.3543	0.7233	-0.6510	0.4523	-0.6558	0.5257
	OAW.cons.moll	1	0.3969	0.4888	0.8119	0.4174	-0.5646	1.3584	NA	NA
	OAW.cons.phyto	8	-0.3186	0.1922	-1.6571	0.0984	-0.6967	0.0596	**-0.9656**	**-0.0055**
	OAW.cons.zoop	6	-0.3040	0.2187	-1.3897	0.1656	-0.7342	0.1263	-0.9752	0.2540
	OAW.res.algae	6	0.0415	0.2906	0.1427	0.8866	-0.5303	0.6132	-0.2089	0.5130
	OAW.res.CCA	1	0.8938	0.6161	1.4508	0.1478	-0.3181	2.1057	NA	NA
	OAW.res.macro	18	0.0669	0.1932	0.3460	0.7295	-0.3132	0.4470	-0.1941	0.2455
	OAW.res.moll	2	-0.6168	0.5831	-1.0578	0.2909	-1.7639	0.5303	NA	NA
	OAW.res.plant	1	-0.1157	0.6813	-0.1698	0.8652	-1.4561	1.2246	NA	NA
	W.both.CCA	2	0.1873	0.3927	0.4770	0.6337	-0.5852	0.9598	NA	NA
	W.both.cnid	1	0.0662	0.5880	0.1126	0.9104	-1.0905	1.2229	NA	NA
	W.both.crust	3	0.0273	0.4082	0.0669	0.9467	-0.7756	0.8302	**0.0160**	**0.1779**
	W.both.echin	2	-0.8987	0.7218	-1.2451	0.2140	-2.3187	0.5212	NA	NA
	W.both.fish	2	0.4108	0.4024	1.0207	0.3081	-0.3809	1.2025	NA	NA
	W.both.macro	12	0.1328	0.1977	0.6715	0.5023	-0.2562	0.5217	-0.0546	0.2425
	W.both.moll	5	-0.1320	0.2879	-0.4584	0.6470	-0.6984	0.4344	**-0.5213**	**-0.0093**
	W.both.plant	4	-0.7263	0.3912	-1.8565	0.0643	-1.4958	0.0433	**-1.9815**	**-0.1614**
	W.both.turf	4	0.0710	0.3002	0.2364	0.8133	-0.5196	0.6615	-0.8164	0.5260
	W.cons.feed	12	0.1989	0.1718	1.1581	0.2476	-0.1390	0.5369	**0.0864**	**0.3785**
	W.cons.macro	11	-0.1473	0.2357	-0.6249	0.5325	-0.6109	0.3164	-0.4817	0.1909
	W.cons.moll	2	0.2998	0.4454	0.6733	0.5012	-0.5763	1.1760	NA	NA
	W.cons.phyto	9	-0.1247	0.1812	-0.6882	0.4918	-0.4811	0.2317	-0.5706	0.2549
	W.cons.plant	2	-0.1776	0.4079	-0.4354	0.6635	-0.9800	0.6248	NA	NA
	W.cons.zoop	10	0.0948	0.1833	0.5174	0.6052	-0.2658	0.4554	-0.1318	0.4827
	W.res.CCA	1	0.3566	0.6075	0.5869	0.5577	-0.8386	1.5517	NA	NA
	W.res.macro	30	0.0871	0.1827	0.4765	0.6340	-0.2723	0.4465	-0.1595	0.2747
	W.res.moll	2	-0.2429	0.6928	-0.3505	0.7262	-1.6057	1.1200	NA	NA
	W.res.plant	1	0.2904	0.7969	0.3644	0.7158	-1.2773	1.8581	NA	NA

Treatment × Trophic Role × Life Stage$_{cons}$	OA.both.adult	30	0.1435	0.1240	1.1571	0.2486	-0.1010	0.3879	**0.0519**	**0.3699**
	OA.both.juv	7	0.2385	0.2285	1.0440	0.2977	-0.2119	0.6890	**0.0635**	**0.4030**
	**OA.cons.adult**	**33**	**-0.3030**	**0.1045**	**-2.9011**	**0.0041**	**-0.5090**	**-0.0971**	**-0.3945**	**-0.0783**
	OA.cons.juv	27	-0.0589	0.1064	-0.5532	0.5807	-0.2687	0.1509	-0.1527	0.0372
	OA.cons.larv	22	-0.1817	0.1271	-1.4295	0.1544	-0.4322	0.0689	**-0.5439**	**-0.0705**
	OA.res.adult	10	-0.0109	0.1692	-0.0646	0.9486	-0.3446	0.3227	-0.3435	0.1123
	OA.res.juv	*2*	*0.6880*	*0.3220*	*2.1366*	*0.0338*	*0.0531*	*1.3229*	*NA*	*NA*
	OAW.both.adult	11	0.2435	0.1714	1.4206	0.1570	-0.0945	0.5815	-0.0078	0.4535
	OAW.both.juv	*2*	*0.2285*	*0.3164*	*0.7221*	*0.4711*	*-0.3953*	*0.8522*	*NA*	*NA*
	**OAW.cons.adult**	**9**	**-0.6946**	**0.1750**	**-3.9690**	**0.0001**	**-1.0397**	**-0.3496**	**-1.1255**	**-0.1870**
	OAW.cons.juv	10	0.0348	0.1458	0.2385	0.8117	-0.2527	0.3222	-0.1619	0.3853
	OAW.cons.larv	4	-0.1247	0.2345	-0.5316	0.5956	-0.5870	0.3376	**-2.6494**	**-0.3030**
	OAW.res.adult	4	0.2145	0.2828	0.7584	0.4491	-0.3431	0.7720	-0.3906	0.7554
	W.both.adult	13	0.1392	0.1761	0.7904	0.4302	-0.2080	0.4863	**0.0083**	**0.3276**
	W.both.juv	3	0.1378	0.2816	0.4895	0.6250	-0.4173	0.6930	**0.0290**	**0.3318**
	W.cons.adult	9	-0.0265	0.1709	-0.1549	0.8771	-0.3635	0.3106	-0.1234	0.3909
	W.cons.juv	14	0.0544	0.1374	0.3959	0.6926	-0.2165	0.3253	-0.1902	0.3316
	W.cons.larv	6	0.0204	0.1857	0.1101	0.9125	-0.3457	0.3866	-0.8560	0.5669
	W.res.adult	9	-0.0330	0.2517	-0.1312	0.8957	-0.5293	0.4632	-0.5572	0.2011
	OA.both.adult	*1*	*0.2020*	*0.4897*	*0.4124*	*0.6820*	*-0.7838*	*1.1877*	*NA*	*NA*
Treatment × Trophic Role × Life Stage$_{res}$	OA.both.juv	7	-0.0144	0.1920	-0.0751	0.9404	-0.4009	0.3720	**0.0137**	**0.3465**
	OA.both.larv	12	0.2827	0.1745	1.6198	0.1121	-0.0686	0.6340	-0.1268	0.3294
	OA.cons.adult	3	-0.4009	0.2594	-1.5453	0.1291	-0.9231	0.1213	-1.0670	0.0376
	**OA.cons.juv**	**13**	**-0.5406**	**0.1678**	**-3.2213**	**0.0023**	**-0.8785**	**-0.2028**	**-0.9248**	**-0.2651**
	OA.res.juv	6	0.1276	0.2613	0.4885	0.6275	-0.3983	0.6535	**0.0691**	**0.4363**
	OAW.both.adult	*1*	*-0.2535*	*0.4752*	*-0.5334*	*0.5963*	*-1.2101*	*0.7031*	*NA*	*NA*
	**OAW.both.larv**	**3**	**0.5409**	**0.2387**	**2.2662**	**0.0282**	**0.0605**	**1.0213**	**0.2568**	**0.7657**
	OAW.cons.adult	*2*	*-1.3839*	*0.3654*	*-3.7868*	*0.0004*	*-2.1195*	*-0.6482*	*NA*	*NA*
	OAW.res.adult	*1*	*0.1014*	*0.4411*	*0.2299*	*0.8192*	*-0.7865*	*0.9894*	*NA*	*NA*
	OAW.res.juv	*2*	*-0.6754*	*0.4813*	*-1.4033*	*0.1672*	*-1.6442*	*0.2934*	*NA*	*NA*
	W.both.adult	*2*	*0.5660*	*0.3104*	*1.8234*	*0.0747*	*-0.0588*	*1.1907*	*NA*	*NA*
	W.both.juv	*2*	*-0.3435*	*0.2629*	*-1.3069*	*0.1977*	*-0.8726*	*0.1856*	*NA*	*NA*
	W.both.larv	3	0.3038	0.2463	1.2335	0.2236	-0.1919	0.7996	-0.0142	0.5510
	W.cons.adult	*1*	*0.1005*	*0.4653*	*0.2160*	*0.8299*	*-0.8362*	*1.0372*	*NA*	*NA*
	W.cons.juv	*1*	*1.6076*	*1.0766*	*1.4932*	*0.1422*	*-0.5594*	*3.7746*	*NA*	*NA*
	W.cons.larv	*2*	*0.6731*	*1.1911*	*0.5651*	*0.5747*	*-1.7244*	*3.0707*	*NA*	*NA*
	W.res.juv	*2*	*-0.2934*	*0.6101*	*-0.4809*	*0.6329*	*-1.5213*	*0.9346*	*NA*	*NA*
Treatment (choice)	OA	10	-0.1451	0.2947	-0.4925	0.6291	-0.7699	0.4796	-0.6812	0.0707
	OAW	3	-0.4747	0.4081	-1.1631	0.2619	-1.3399	0.3905	-0.4228	0.0231
	W	6	-0.7150	0.3645	-1.9618	0.0674	-1.4877	0.0576	-0.7184	0.0637
NA (Prey survival)	**OA**	**7**	**-1.8119**	**0.4275**	**-4.2382**	**0.0055**	**-2.8581**	**-0.7658**	**-2.2091**	**-0.9005**

Table S3. Summary statistics of Omnibus tests of moderators and heterogeneity from models of consumption from fully factorial studies. Moderators tested were *Consumption Type* (predation or grazing) and the environmental variables, ocean acidification and/or warming (*Treatment*). The lower table provides the model estimates (mean ln RR) per treatment.

			Omnibus test				Heterogeneity					
Response	Moderator	k	F	df_m	df_{k-p}	p-value	$QE_{df\,(k-p)}$	$QE_{p\text{-value}}$	$\sigma^2_{(btw)}$	$\sigma^2_{(w/in)}$	$I^2_{(btw)}$	$I^2_{(w/in)}$
Consumption	Consumption Type × Treatment	199	0.6741	5	193	0.6436	1515.7	<0.0001	0.1802	0.1269	54.232	38.209
	Consumption Type + Treatment	199	0.7803	3	195	0.5063	1531.5	<0.0001	0.1823	0.1250	54.839	37.605
	Consumption Type	199	0.2308	1	197	0.6315	1606.4	<0.0001	0.1817	0.1270	54.437	38.040
	Treatment	199	1.0540	2	196	0.3505	1532.0	<0.0001	0.1759	0.1252	53.928	38.375

				Coefficients				95% CI	
	Moderator	Moderator level	k	estimate	SE	t-value	p-value	lower	upper
Consumption	Treatment	OA	62	0.01794	0.09276	0.1934	0.8468	-0.1650	0.2009
		W	69	0.11155	0.09207	1.2116	0.2271	-0.0700	0.2931
		OAW	68	0.00815	0.09111	0.0894	0.9288	-0.1715	0.1878

Table S4. Summary of data diagnostics. Three-level models of k studies were fit without moderators using restricted maximum-likelihood estimation (REML) and included *Effect Size* nested within *Publication* as random effects. Potential outliers were removed from each dataset and models were reassessed. Summary statistics of the variance components consist of the residual heterogeneity (QE), between-publication variance ($\sigma^2_{between}$) which is equivalent to τ^2, within-publication variance (σ^2_{within}), and the distribution of variance (%) across levels ($I^2_{between}$ and I^2_{within}). Model coefficients were based on a t-distribution and significant effect sizes ($p<0.05$) are in bold. Publication bias was assessed with Egger's regression tests, with significant coefficients reported indicating asymmetry in funnel plots.

			Heterogeneity				Sensitivity analysis			
Response	k	outlier	QE	$QE_{p\text{-}value}$	$\sigma^2_{(btw)}$	$\sigma^2_{(w/in)}$	estimate	SE	t-value	p-value
Consumption	389	0	5054.0	<0.0001	0.1593	0.1243	-0.0510	0.0458	-1.1136	0.2661
	388	1	4936.1	<0.0001	0.1519	0.1228	-0.0418	0.0452	-0.9242	0.3560
Grazing (choice)	20	0	156.41	<0.0001	<0.0001	0.6491	-0.3586	0.1969	-1.8216	0.0843
	19	1	122.51	<0.0001	0.1222	0.2215	-0.2796	0.2113	-1.3233	0.2023
Prey survival	9	0	23.192	0.0031	<0.0001	1.5861	-1.9718	0.5351	-3.6847	0.0062
	8	1	12.6025	0.0824	<0.0001	0.5167	-1.5755	0.4141	-3.8044	0.0067

			Egger's regression test			
Response	k	outlier	estimate	SE	t-value	p-value
Consumption	389	0	-0.2680	0.1908	-1.4047	0.1609
	388	1	-0.2828	0.1897	-1.4913	0.1367
Grazing (choice)	20	0	-2.2634	1.1568	-1.9566	0.0661
	19	1	-1.1100	0.9569	-1.1600	0.2621
Prey survival	9	0	-3.1619	1.8742	-1.6870	0.1355
	8	1	-1.9143	1.6084	-1.1902	0.2789

Dataset legends:

Dataset S1 (.xlsx). Completed RepOrting standards for Systematic Evidence Syntheses(ROSES) checklist of systematic review protocols (2).

Dataset S2 (.xlsx). Database of response and metadata extracted from studies, calculated effect sizes, and data diagnostics.

Dataset S3 (.csv). Dataset of studies that measured consumption rates and were included in the corresponding analyses.

Dataset S4 (.csv). Dataset of studies that measured consumption rates in choice experiments and were included in the corresponding analyses.

Dataset S5 (.csv). Dataset of studies that measured prey survival and were included in the corresponding analyses.

Dataset S6 (.txt). R code used to conduct all analyses.

SI References

1. N. R. Haddaway, B. Macura, P. Whaley, A. S. Pulli, ROSES flow diagram for systematicreviews Version 1.0 (2017). 10.6084/m9.figshare.5897389

2. N. R. Haddaway, B. Macura, P. Whaley, A. S. Pulli, ROSES for Systematic ReviewProtocols. Version 1.0 (2017). 10.6084/m9.figshare.5897269
